# Targeting IL-2: an unexpected effect in treating immunological diseases

**DOI:** 10.1038/s41392-017-0002-5

**Published:** 2018-01-19

**Authors:** Congxiu Ye, David Brand, Song G. Zheng

**Affiliations:** 1 Department of Clinical Immunology, Third Affiliated Hospital at Sun Yat-sen University, Guangzhou, China; 2 Research Service, Memphis VA Medical Center, Memphis, TN USA; 3 Division of Rheumatology, Penn State Milton S. Hershey Medical Center, Hershey, PA USA

## Abstract

Regulatory T cells (Treg) play a crucial role in maintaining immune homeostasis since Treg dysfunction in both animals and humans is associated with multi-organ autoimmune and inflammatory disease. While IL-2 is generally considered to promote T-cell proliferation and enhance effector T-cell function, recent studies have demonstrated that treatments that utilize low-dose IL-2 unexpectedly induce immune tolerance and promote Treg development resulting in the suppression of unwanted immune responses and eventually leading to treatment of some autoimmune disorders. In the present review, we discuss the biology of IL-2 and its signaling to help define the key role played by IL-2 in the development and function of Treg cells. We also summarize proof-of-concept clinical trials which have shown that low-dose IL-2 can control autoimmune diseases safely and effectively by specifically expanding and activating Treg. However, future studies will be needed to validate a better and safer dosing strategy for low-dose IL-2 treatments utilizing well-controlled clinical trials. More studies will also be needed to validate the appropriate dose of IL-2/anti-cytokine or IL-2/anti-IL-2 complex in the experimental animal models before moving to the clinic.

## Immunotherapy: Interleukin-2 therapy promising for autoimmune disease

Low doses of the immune signaling molecule interleukin-2 (IL-2) could help suppress unwanted immune responses in patients with autoimmunity. Song Zheng from Penn State Health Milton S. Hershey Medical Center, USA and Sun Yat-sen University, and colleagues reviewed the key role that IL-2 plays in the development and function of regulatory T cells, a type of suppressive immune cell that keeps the rest of the immune system in check. The researchers summarized data from proof-of-concept clinical trials, which showed that, at low doses, IL-2 is well tolerated and induces regulatory T cells to address immune imbalances in patients with lupus, type 1 diabetes and other autoimmune disorders. However, the researchers suggest that IL-2 treatments might need to be combined with other immune-modulating strategies to maximize the therapeutic benefit while minimizing side effects.

## Introduction

Regulatory T cells (Treg) are crucial in maintaining immune homeostasis and prevention of autoimmune diseases.^1,2^ Treg cells are generally divided into two major subsets: naturally occurring, thymus-derived cells that react to self-antigens (Ags) and peripherally, or ex vivo induced, adaptive Treg cells that primarily respond to environmental Ag or in vitro stimulation with cytokine TGF-β.^3–7^ Most of these cells are CD4^+^ cells that express CD25 (interleukin-2 (IL-2) receptor alpha chain) and the fork head box protein P3 (Foxp3) transcription factor that is required for their differentiation and function.^8–11^

When first discovered in 1976, IL-2 was characterized as a soluble factor with the unique ability to promote clonal expansion of T cells in vitro.^12^ It is primarily produced by antigen-activated T cells and can bind to three different high-affinity receptor subunits on target cell membranes.^13^ The discovery made by our group and others that IL-2 is a key cytokine for Treg cell differentiation, survival, and function^14–17^ has led to new opportunities for tipping the balance between Treg and effector T cells towards Tregs. Furthermore, clinical trials using low-dose IL-2 have demonstrated that IL-2 shows great potential for expanding Treg cells and modulating immune pathologies.^18–29^

Here we review the current knowledge about IL-2 and its receptors, discuss the relationship between low-dose IL-2 and Treg development, especially focusing on mechanistic studies and clinical trials. These studies and trials have led to a shift in our understanding of IL-2 from a cytokine known for the activation of effector T cells against cancer when used at a high dose, to a cytokine that activates Treg cells to control autoimmunity at a low dose.

## IL-2 biology

**Fig. 1:**
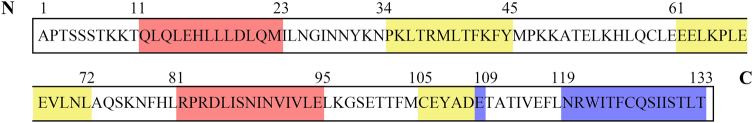
Model structure of IL-2. Human IL-2 comprises 133 amino acids and weighs 15.5 kDa, while mouse IL-2 is comprised of 149 amino acids and weighs 16 kDa; they show 57% sequence homology. The amino acids that interact with IL-2R subunits are designated in yellow for IL-2Rα, red for IL-2Rβ and blue for γ_c_

IL-2, also named T-cell growth factor, was first discovered in 1976 and was characterized as a soluble factor with the unique ability to promote clonal expansion of T cells in vitro.^12^ IL-2 is a 15.5 kDa four-bundle, α-helical protein member of the common cytokine receptor γ-chain family of cytokines (Fig. 1). First cloned in 1983, it has been the most highly investigated IL with a diverse role in the regulation of the immune system.^30^ IL-2 is predominantly produced by activated CD4^+^ T cells and, to a lesser extent by activated CD8^+^ T cells, activated dendritic cells, natural killer (NK) cells, NKT cells, as well as B cells.^31^

**Fig. 2:**
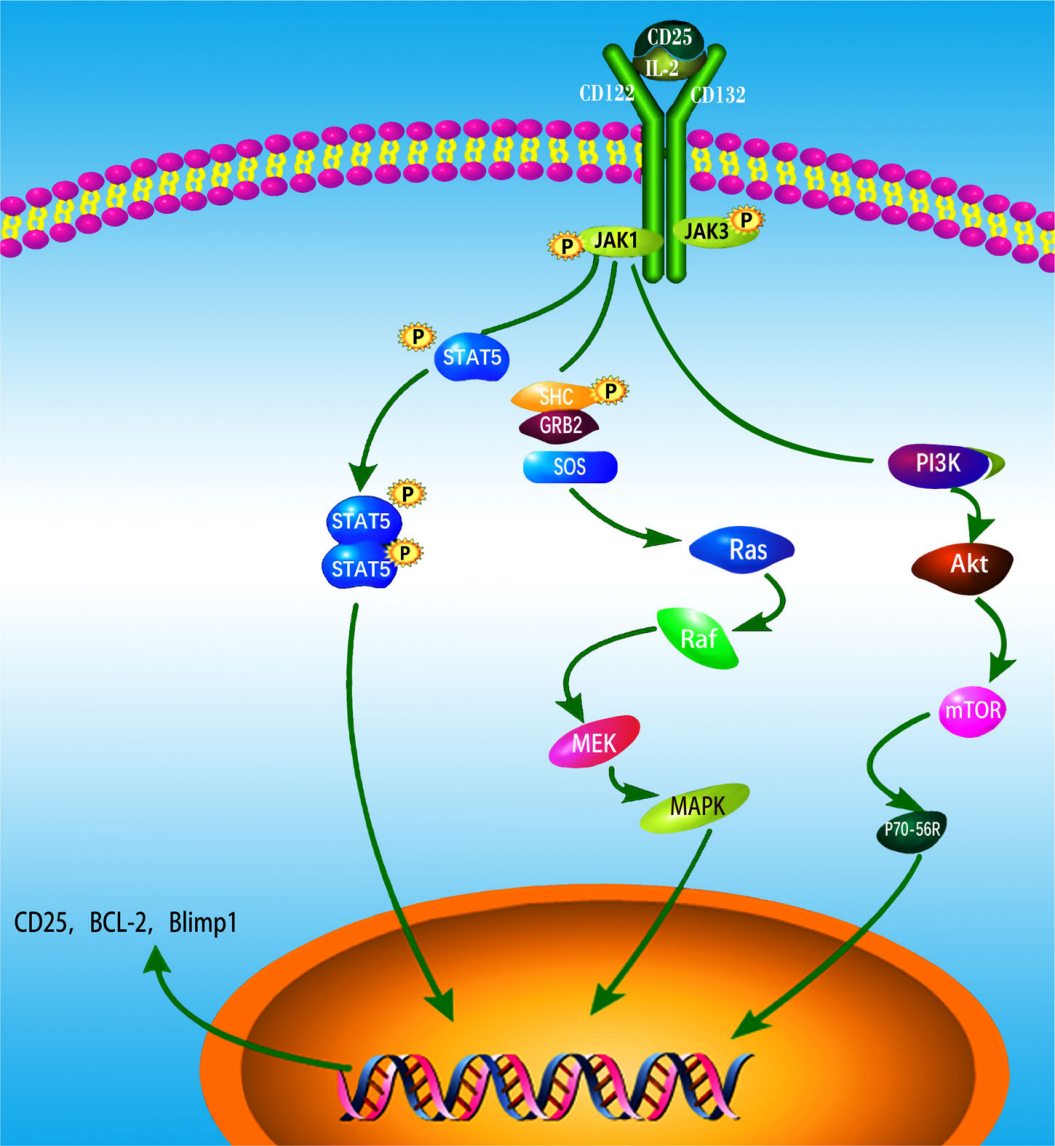
IL-2 binding to IL-2 receptors (IL-2Rs) and downstream signaling. The high-affinity IL-2 receptor is comprised of CD25 (IL-2Rα), CD122 (IL-2Rβ), and CD132 (IL-2Rγ_c_). The signaling function of the high-affinity IL-2 receptor is mediated through IL-2Rβ and γ_c_ chains. These chains constitutively bind to JAK1 and JAK3 and phosphorylate which then recruit STAT5a/STAT5b via these molecules’ SH2 domains. Phosphorylation of STAT5a/5b by JAKs results in the heterodimeric complex, which regulates gene transcription by binding to target DNA sequences. Phosphorylation of the adaptor SHC also leads to the activation of the Ras–Raf–MAPK and PI3K pathways. Any one or all of the STAT5a/b, Ras–Raf–MAPK, and PI3K signaling pathways can be activated under the appropriate situation. This accounts for IL-2’s pleiotropic actions on target cells

IL-2 exerts its pleiotropic biological activities in autocrine or paracrine fashion by binding to its receptors, which consists of three subunits including IL-2Rα(CD25), IL-2Rβ(CD122), and IL-2Rγ_c_(CD132).^13^ The heterotrimeric association of the IL-2Rα chain with IL-2Rβ and IL-2Rγ_c_ provides a high-affinity receptor for IL-2^32^. The heterodimeric association of the IL-2Rβ chain and the common γ chain forms the intermediate-affinity IL-2R (binding affinity Kd ≈ 1 nM), present in resting T cells, memory CD8^+^ T cells and NK cells.^14^ By contrast, the IL-2Rαβγ_c_ heterotrimeric complex, also known as the high-affinity IL-2R receptor (Kd ≈ 10 pm), is transiently expressed by activated immune cells, including T and B lymphocytes, NK cells, and DCs even though it is constitutively expressed on Foxp3-expressing CD4^+^ Tregs.^33^ CD25 is a membrane protein with extensive N- and O-linked glycosylation that contains a short cytoplasmic tail lacking both kinase activity and phosphotyrosine motifs and which therefore does not have intrinsic signaling properties. The signaling function of the high-affinity IL-2 receptor is mediated through the β and γ chains. While these chains also lack kinase activity, they constitutively bind to the Janus kinases JAK1 and JAK3. JAK1 and JAK3 kinase binding initiates phosphorylation of the β and γ chains, respectively, which then recruit STAT5a/STAT5b via their SH2 domains. STAT5a/5b phosphorylation results in their translocation into the nucleus as a heterodimeric STAT complex, regulating gene transcription through binding to target DNA sequences.^34,35^ Alternatively, IL-2 signaling can induce the phosphorylation of the adaptor protein Shc which activates Ras–Raf MAP kinase and PI-3K pathways.^36^ Altogether, STAT5a/b, Ras–Raf MAP kinase and PI-3K signaling pathways allow rapid transmission of IL-2 signals from the membrane to the nucleus. These signals control, in a direct and indirect manner, the expression and function of multiple master regulator transcription factors implicated in several differentiation and immune regulatory pathways^37^ (Fig. 2).

**Fig. 3:**
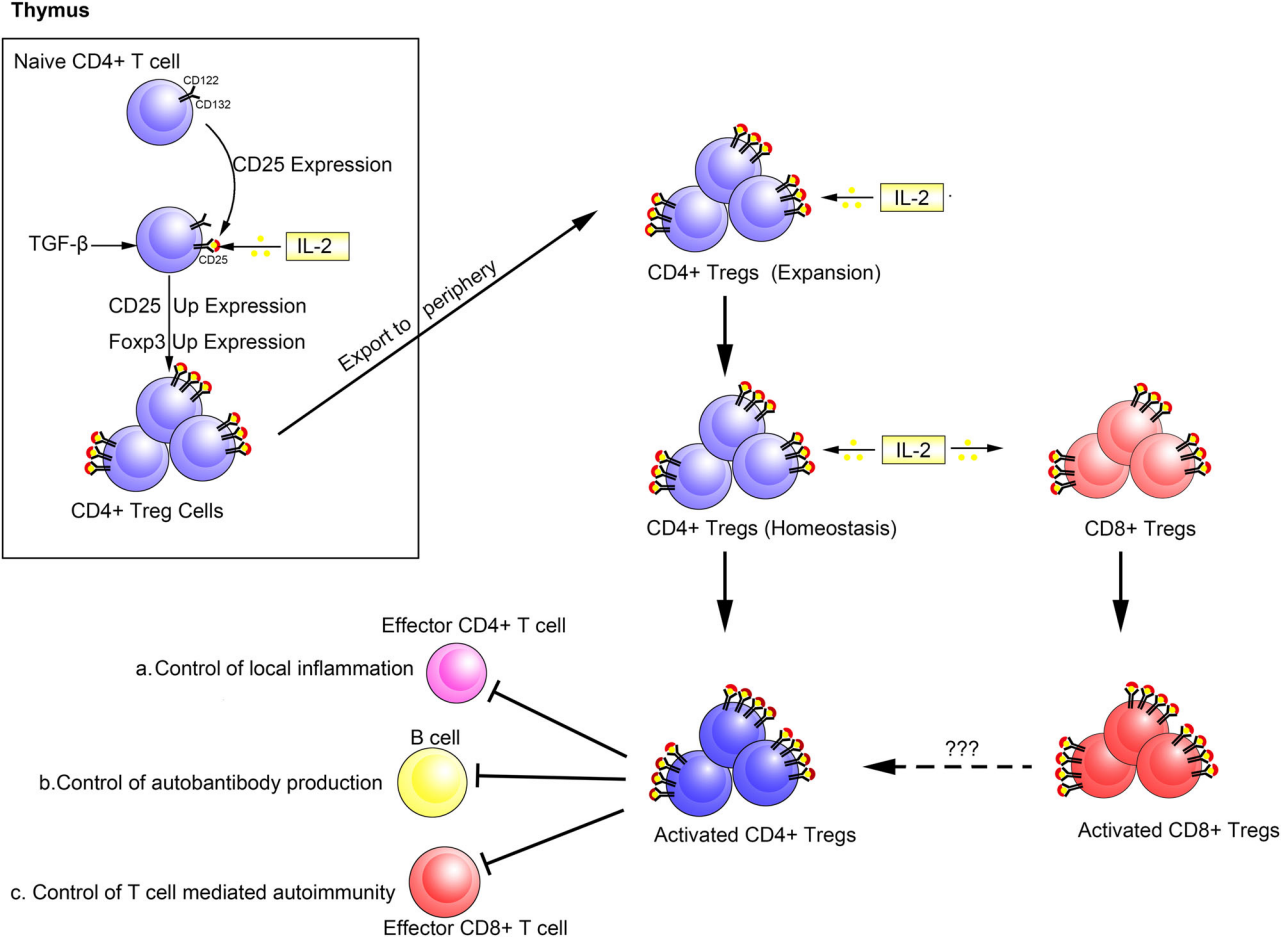
Main role of IL-2 signal in thymus and in peripheral Tregs. In the thymus, naive CD4^+^ T cells transiently express CD25 and respond to IL-2 through the STAT5s signaling pathway, and up regulate CD25 and Foxp3 expressions. Then, they begin to differentiation into mature CD4^+^ Tregs. Mature CD4^+^ Tregs emigrate from the thymus and constitutively express CD25 and respond vigorously to IL-2 by up regulating CD25 and becoming activated CD4^+^ Tregs. CD8^+^ T cells also respond to IL-2 and become activated CD8^+^ Tregs. Activated CD4^+^ Tregs exert their suppressive functions to include **a** control of local inflammation; **b** control of autoantibody production; (**c**) control of T-cell-mediated autoimmunity. However, the mechanisms of the suppressive activity of CD8^+^ Tregs in vivo remain to be determined

IL-2 is a pleiotropic cytokine with myriad functions. Where Tregs are concerned, it supports their development in the thymus^38^ (Fig. 3), is a key survival factor for them in the periphery, and is required for their functional competence and stability^17,39,40^ (Fig. 3).

## IL-2 and tregs

### IL-2 is Involved in Treg Development, Stability, and Function

Supporting the initial hypothesis that IL-2 is required for Treg development in vivo, work from Malek et al.^41^ demonstrated that the autoimmune disease expressed in Il2rb^−^^/−^ mice could be prevented through transfer of CD25^+^ Tregs from WT mice into Il2rb^−^^/−^ host mice. These studies demonstrated that Il2rb^−^^/−^ mice lacked a functional population of Tregs. Additional work by this group demonstrated that thymus-specific expression of an Il2rb transgene was sufficient to rescue the defect in Tregs development suggesting that the defect in Il2rb^−^^/−^ mice is due to a failure of Treg development in the absence of IL-2.^42^

Our research group made the first observation that IL-2 is important for the differentiation and function of CD4^+^ Foxp3^+^ Treg induced ex vivo.^17^ This finding was immediately validated by the laboratory of Dr. Ethan Shevach.^43^ Although TGF-β and its receptor signal is essential for the Treg induction and development (induced Treg, iTreg)^5^ and its functional characteristics have been widely studied^44–48^, the role IL-2 plays in iTreg development, stability, and function is by no means redundant.^17,43^

### IL-2 signal plays a major role in thymic treg differentiation

The importance of IL-2R signaling in thymic Treg differentiation is clearly demonstrated by the fact that the lethal autoimmunity in mice lacking Il2rb is due to a failure to generate thymic Tregs, and this phenotype is completely restored by adoptive transfer of small numbers of wild-type Tregs.^41^ Moreover, retroviral transduction of Il2rb^−^^/−^ bone marrow with wild-type Il2rb, or a mutant construct capable of activating only STAT5 via Tyr-510, restored thymic Treg generation in bone marrow of chimeric mice. In contrast, restoration of Treg development did not occur when mutant constructs capable of activating RAS/PI3K, but not STAT5, were transduced into Il2rb^−^^/−^ bone marrow cells and engrafted into recipient mice.^49^ Likewise, crossing Il2rb^−^^/−^ mice to transgenic mice expressing a constitutively active form of STAT5b (STAT5b-CA mice)-restored Treg development in the thymus.^49^ Additional support for the role of STAT5 in Treg development came from two studies which demonstrated that conditional deletion of STAT5 in DP thymocytes (i.e., CD4-Cre × STAT5a/b^FL/FL^ mice) had minimal effects on CD4SP thymocytes with the exception of CD4^+^ Foxp3^+^ thymic Treg.^49,50^ Altogether, these findings indicate that STAT5 activation on downstream of IL-2R is required for thymic Treg development.

### 2.3 The IL-2 signal plays a major role in the homeostasis and function of treg cells

There is an abundance of evidence demonstrating that the IL-2 signal plays a major role in the homeostasis and activation of CD4^+^ CD25^+^ Foxp3^+^ Tregs.^15^ The quaternary complex consisting of IL-2 and IL-2Rα/β/γ induces the phosphorylation of STAT5, resulting in increased expression of CD25 and Foxp3 on Treg and activation of their suppressive activity.^15,51^ In the absence of IL-2 signaling, there was approximately a 50% reduction in the absolute numbers of Foxp3-expressing Treg cells in the thymic CD4 single-positive subset.^39^ The absolute numbers of Foxp3^+^ T cells in the periphery, however, were similar to that seen in wild-type mice, presumably because lymphopenia-induced lymphproliferation of the depleted thymic Treg pool was able to overcome the Treg developmental defect.^39^ Another study followed the fate of a hemaglutinin-specific monoclonal T-cell population in IL-2-deficient, HA/TCR-double transgenic mice. The data suggested that IL-2/IL-2R signaling is necessary for the peripheral maintenance, but not development, of Foxp3^+^ Treg cells.^52^ This conclusion was supported, in part, by the observations of another study reporting that IL-2/IL-2R signaling is required for Treg cell proliferation in vivo.^53^ In contrast, studies following the fate of HEL-specific T cells in IL-2-deficient mice expressing HEL under control of an insulin promoter^54^ were more consistent with the idea that IL-2/IL-2R signaling is indispensable for development of Foxp3^+^ Treg cells. Takatoshi Chinen et al.^55^ found that the capture of IL-2 was dispensable for control of CD4^+^ T cells but was important for limiting the activation of CD8^+^ T cells, and that IL-2R-dependent activation of the transcription factor STAT5 had an essential role in the suppressor function of Treg cells separable from signaling via the T-cell antigen receptor by using genetic gain- and loss-of-function approaches. They hypothesized that genetic modification of Treg cells might hold promise for the optimal design of Treg cell-based therapies for a variety of autoimmune and inflammatory disorders and organ transplantation. When taken together, these observations in neo-antigen-specific TCR-transgenic mice suggest that differences in the levels and/or anatomical geography of antigen expression and/or in the affinities of the transgenic TCRs under study influence the extent with which IL-2/IL-2R deficiencies affect Treg development, peripheral homeostasis, or function.^56^

Notwithstanding these discrepancies, several lines of evidence support the idea that IL-2/IL-2R signaling is necessary for peripheral homeostasis of the CD4^+^ CD25^+^ Treg population. First, the peripheral CD4^+^ CD25^+^ Treg pool is significantly reduced or absent in the absence of IL-2 or IL-2R.^39,52,53^ Second, reconstitution of IL-2-competent hosts with a mixture of wild-type and IL-2R-deficient bone marrow revealed that peripheral Treg arise exclusively from the wild-type donor marrow.^57^ Third, the size of the peripheral CD4^+^ CD25^+^ Treg population in neonatal mice was significantly reduced upon administration of neutralizing anti-IL-2 antibodies.^52^ Finally, treatment of NOD mice with a neutralizing anti-IL-2 antibody compromised Treg immune regulation and resulted in the development of accelerated T1D.^58^ Research has demonstrated that IL-2-deficient and IL-2R-deficient mice can be found to contain Foxp3^+^ CD25^−^ T cells which would suggest that Treg development is not IL-2 dependent.^39,52^ These cells, however, express lower levels of Foxp3 and represent immature, non-functional Treg cells.^57^ Yet another study along these lines found that IL-2^−^^/−^Bim^−^^/−^ mice contain normal numbers of Foxp3^+^ cells in the periphery, but these cells also were not functional and the mice developed a lethal autoimmune phenotype.^59^

#### IL-2-deficient, IL-2R-deficient, or STAT5-deficient models and treg cells

In early phenotypic analyses of IL-2-deficient mice, Schorle and Sadlack found that lack of IL-2 does not lead to immunodeficiency or a failure of peripheral T cells to get activated but rather to hyper activation of the CD4 T-cell compartment.^60,61^ Very similar phenotypes were also seen in IL-2Rβand IL-2Rα deficient mice.^62,63^ Further research showed that deletion of IL-2 resulted in the absence of CD4^+^ CD25^+^ Tregs, which resulted in the occurrence of T-cell-mediated autoimmune diseases.^58^ Barmeyer et al. found that T cells and NK cells were present in neonatal mice with the deletion of IL-2.^64^ At 4 weeks, hyperplasia of lymph node, intestinal lymphoid tissue and splenomegaly occurred, at the same time the concentration of autoantibody in peripheral blood increased.^64^ At 9 weeks, 25–50% mice died of severe hemolytic anemia, while others suffered from severe inflammatory bowel diseases and other autoimmune phenotypes.^64^ Malek et al.^65^ also showed that the absence of Treg development in IL-2-and IL-2R-deficient mice resulted in a lymphoproliferative autoimmune syndrome. Similarly, autoimmune disease development is linked to IL-2 deficiencies in humans^66–68^, most likely due to the expansion of immature non-functional Treg in the absence of IL-2 signals.^69,70^

Mice that lack STAT5a and STAT5b display massive lymphocyte infiltration of multiple organs.^71^ In these mice, there is a dramatic reduction in the size of the CD4^+^ CD25^+^ Treg population, resembling that seen in IL-2Rα-deficient mice. This is likely due to lack of Foxp3 expression, which requires STAT5, one of the key signal transduction molecules that connect IL-2 receptor activation to gene regulation in the nucleus.^72,73^

On the basis of these findings described above, it is likely that the appropriate low dose of IL-2 could be used to simulate Tregs and then in directly to reduce Th17 and Tfh activity. IL-2 might indeed become a prime candidate for the treatment of a broad range of autoimmune diseases.

#### Low-dose IL-2 treats diseases through expansion of treg cells

Base on the discovery that IL-2 is a key cytokine for Treg cell differentiation, survival and function, many studies tried to find out the therapeuticy potential in animal models and humans. Yenkel Grinberg-Bleyer et al.^74^ found that 5 days of low-dose IL-2 administration starting at the time of T1D onset can reverse established disease in NOD mice, with long-lasting effects by increasing the numbers of Treg cells in the pancreas and inducing expression of Treg related proteins including Foxp3, CD25, CTLA-4, ICOS, and GITR in these cells. However, Audrey Baeyens et al.^75^ observed that the RAPA/IL-2 combination had a failure to cure T1D. It is possible that RAPA counteracted low-dose IL-2 (25,000 IU daily) and then failed to control IL-2-boosted NK cells, and blocked IL-2-induced tolerance in a reversible way and was associated with an unexpected deleterious effect on glucosehomeostasis at multiple levels, including B-cell division, glucose tolerance, and liver glucose metabolism. In 2016, Benjamin Bonnet et al.^76^ discovered that low-dose IL-2 (ld-IL-2)-induced Treg expansion and activation that elicited protection against clinical manifestations of food allergy in two mouse models with OVA and peanut by evaluating the ability of ld-IL-2 to control allergy in an experimental model of food allergy, and the preventive and therapeutic effects were long-term effective over a 7-month period. Their further mechanistic studies indicated that protection from allergy could be explained by a Treg-dependent local modification of the Th1/Th2 balance and an inhibition of mast cell recruitment and activation. This study demonstrated that ld-IL-2 is efficient to prevent and to treat allergic immune responses, and thus represents a promising therapeutic strategy for managing allergic diseases for human in future.

Therapeutic use of IL-2 in humans was initially used at high doses. Ahmadzadeh et al.^77^ studied the impact of IL-2 administration on the frequency and function of human CD4^+^ CD25^hi^ T cells in immune intact patients with melanoma or renal cancer, and found that administration of high-dose IL-2 increased the frequency of circulating CD4+ CD25hi Foxp3+ regulatory T cells, its suppressive activity and the expression of phenotypic markers associated with regulatory T cells, such as Foxp3. However, more and more clinical studies found that high-dose IL-2 has a poor safety profile but a robust efficacy in only a fraction of patients with a restricted set of cancers. And the high toxicity severely restrained the use of high-dose IL-2.

In recent years, it has been found that to avoid the severe side effects of high-dose IL-2 and also prevent, as far as possible, the activation of effector T cells, using low-dose IL-2 could be the solution. So, in this part, we summarize the clinical trials of low-dose IL-2 treatment in autoimmune diseases.

### Low-dose IL-2 treatment and hematopoietic stem cell transplantation (HSCT)

Zorn et al. treated chronic myelogenous leukemia patients after allogeneic hematopoietic stem cell transplantation (HSCT) with low-dose IL-2 and found that treatment resulted in a 1.9 median fold increase in the numbers of CD4^+^ CD25^+^ cells in peripheral blood as well as a 9.7 median fold increase in Foxp3 expression in CD3^+^ T cells, and that this effect appeared to increase as the treatment condition.^72^ In 2009, the same group also found that the combination of low dose IL-2 with Treg infusion can significantly improve the clinical effect of HSCT by increasing the numbers of Treg cells in vivo.^18^

### Low-dose IL-2 treatment and HCV-induced vasculitis

Saadoun et al.^19^ evaluated the clinical efficacy and immunologic response after low-dose IL-2 therapy in HCV-related vasculitis patients, and found that treatment resulted in a 3 median folds increase of the numbers of CD4^+^ Treg cells and a 8 median folds increase of CD8^+^ Treg cells in peripheral blood, as well as a small increase in NK and CD56^bright^ NK cells, but decrease of B cells.

### Low-dose IL-2 treatment and T1D

An IL-2 dose determination study was performed in a small cohort of T1D patients in 2013.^20^ This randomized placebo-controlled phase I/II trial was under taken to define the lowest IL-2 dose that could induce Treg cells in 24 adult T1D patients randomized in groups of 6patients to either placebo or IL-2 at doses of 0.33, 1 or 3 million international units per day for a 5-day course. In this study, IL-2 therapy was safe and led to a dose-dependent increase in the numbers of CD4^+^ and CD8^+^ Treg cells with no increase or minimal increase in effector T cells and NK cells, and an absence of deleterious effects on insulin secretion. Rosenzwajg et al. ^21^ also found that expansion of Treg cell populations was accompanied by a marked increase in their expression of activation markers such as CD25, glucocorticoid-induced TNFR-related protein (GITR) and cytotoxic T lymphocyte antigen 4 (CTLA-4). However, both trials were not powered to assess effects on insulin secretion, so further clinical trials enrolling children and adults with recent onset T1D are needed. These studies will be undertaken in Europe (NCT02411253).^78^ Yu et al.^79^ found that IL-2 optimally stimulated tyrosine phosphorylated STAT5 at ~10-fold lower levels of IL-2 than CD45RO^+^ CD4^+^ memory T cells by quantifying the levels of initial proximal signaling and downstream gene activation.

### Low-dose IL-2 treatment and chronic graft-versus-host disease (GVHD)

IL-2 was also shown to have beneficial effects for the treatment of chronic graft-versus-host disease (GVHD), an alloimmune inflammatory disease occurring after allogeneic haematopoietic stem cell transplantation. In a study conducted by Koreth et al.^22^, a cohort of 29 patients with chronic GVHD refractory to glucocorticoid therapy received rIL-2 at three dose levels (300,000, 1 million and 3 million IU) daily for a period of eight weeks. The numbers of CD4^+^ Tregs increased about 8 folds in all patients, and approximately 50% of patients evaluated demonstrated an objective partial response. In another study undertaken to define the mechanisms of action of IL-2 therapy, Matsuoka et al.^23^ examined the immunologic effects of low-dose IL-2 treatment on homeostasis of CD4^+^ T-cell subsets after transplant and found that IL-2 therapy resulted in a selective increase of STAT5 phosphorylation in Tregs and a decrease of pSTAT5 in effector T cells. Over an eight-week period, IL-2 therapy induced a series of changes in Treg homeostasis including increased proliferation and thymic export and an enhanced resistance to apoptosis.^23^ Similarly, low-dose IL-2 administration has been tested in patients receiving hematopoietic stem cell transplantation in order to prevent GVHD.^24^ No adverse events of grade 3 or 4 nor any induction of GVHD were reported. In addition to an expansion of Tregs, viral infections were actually reduced in the low-dose IL-2 treated group compared to the control group.^24^ In this regard, two more studies have been recently completed in chronic GVHD patients.^25,26^ NayounKim et al.^25^ gave a chronic GVHD patient daily low-dose IL-2 treatment for 8 weeks followed by an 18 weeks hiatus and a follow-up administration for another 8 weeks, and demonstrated that low-dose IL-2 could induce a 37.3% expansion of Tregs leading to the improved Treg/Th17 ratios. In another phase II study ^26^, 35 adults with steroid-refractory cGVHD received daily IL-2 treatments of 1 million IU for 12 weeks. Compared with pretreatment levels, Treg and NK cell counts increased over 5-folds and 4-folds, respectively, without significant change in conventional CD4^+^ T cell or CD8^+^ T cell numbers.^26^

### Low-dose IL-2 treatment and alopecia areata

In one study^27^, low-dose recombinant IL-2 induced a marked increase in the numbers of Treg cells accompanied by a marked decrease in the numbers of infiltrating CD8^+^ effector T cells in scalp biopsies of patients with autoimmune alopecia areata during and after IL-2 treatment.

### Low-dose IL-2 treatment and systemic lupus erythematosus (SLE)

In addition to the autoimmune diseases mentioned above, the potential treatment effect of low-dose IL-2 has been further strengthened by success in human clinical studies in systemic lupus erythematosus (SLE). In a study by Humrich et al.^28^, one patient with severe SLE refractory to standard therapy received low-dose IL-2 first at 1.5 MIU and then at 3 MIU for 5-day each. They found that the numbers of Treg increased and the anti-dsDNA antibody levels decreased. To define the effects of low-dose recombinant human IL-2 (rhIL-2) treatment on regulatory and effector CD4^+^ T-cell subsets in patients with active SLE, He et al.^29^ gave 40 patients with active SLE2 MIU IL-2 every other day for 2 weeks followed by a 2-week hiatus, and demonstrated that low-dose IL-2 could both increase the numbers of CD25^high^CD127^low^ Treg cells and enhance their function, and that the ratio of TFH^+^ TH17 cells/Treg cells fell significantly. In addition to clinical trial of low-dose IL-2 above mentioned, there are 14 ongoing clinical trials listed in www.clinicaltrial.org (Table 1) and two modified IL-2 patent applications have been granted (www.uspto.gov).

**Table 1:** Main clinical trials of AID treated with low-dose IL-2

Disease(s)	Year	Trial design	No. patients	Daily IL-2 dose	IL-2 administration schedule	Main findings	Refs
HSCT	2006	Phase I trial?	9	2–4 × 10^5^ U/m^2^ per day	Duration of IL-2 therapy lasted for 4–11 weeks.	(1) Resulted a 1.9 median fold increase in the numbers of CD4^+^ CD25^+^ cells in peripheral blood	^72^
						(2) Resulted a 9.7 median fold increase in Foxp3 expression in CD3^+^ T cells	
						(3) Without clinical toxicities associated with prolonged administration of low-dose IL-2	
HSCT	2009	Phase I trial to investigate the effects of combination of IL-2 and adoptive cellular therapy	5	CD4+ DLI (3 × 10^7^ to 1 × 10^8^ CD4^+^ cells/kg) followed by daily administration of 6 × 10^5^ IU/m^2^ IL-2	12 continuous weeks	(1) Induced peripheral Treg expansion and expanded Treg exhibit normal immune suppressive function	^18^
						(2) Induced a significant increase in FOXP3 gene expression levels	
						(3) IL-2 toxicity in these patients was minimal	
						(4) Significantly improve the medical effect of HSCT	
HCV-related vasculitis	2011	A prospective open-label, phase 1–phase 2a study to investigated the safety and immunologic effects of the administration of low-dose IL-2 in HCV-induced vasculitis patients	10	1.5 then 3 MIU per day	Four 5 days courses	(1) Increased the number of CD4^+^ CD25^high^ FOXP3^+^ Tregs and decreased marginal-zoen B cells	^19^
						(2) Observed one grade 2 and some grade 1 AEs	
						(3) No vasculitis or HCV-replication flares	
						(4) Improvement of the vasculitis in 8/10 patients	
T1D	2011–2012	A phase 1/2 randomized, double-blind, placebo-controlled trial	24	0.33 MIU/day, 1 MIU/day, or 3 MIU/day	one 5-day course	(1) Induced a dose-dependent increase in the proportion of Treg cells	^20^
						(2) IL-2 was well tolerated at all doses, with no serious adverse events. However, there was a dose-response association for non-serious adverse events during the treatment phase	
T1D	2011–2012	A randomized, double-blind, placebo-controlled, dose-finding trial.	24	0.33 MIU/day, 1 MIU/day, or 3 MIU/day	One 5-day course	(1) Induced a dose-dependent increase in CD4+ Foxp3^+^ and CD8^+^ Foxp3^+^ Treg numbers and proportions	^21^
						(2) No serious adverse events were reported during the trial	
Chronic GVHD	2007–2011	A phase 1 dose-escalation study to determine the maximum tolerated dose of daily low-dose subcutaneous IL-2 in patients with active chronic GVHD that was refractory to glucocorticoid treatment.	29	0.3 × 10^6^, 1 × 10^6^, or 3 × 10^6^ IU/m^2^	12-week study assessed daily treatment for 8 weeks, followed by a 4-week hiatus.	(1) The numbers of CD4^+^ Treg cells were preferentially increased in all patients (more than eight times)	^22^
						(2) The Treg:Tcon ratio increased to a median of more than five times	
						(3) The maximum tolerated dose of IL-2 was 1 × 10^6^ IU/m^2^	
						(4) 12 patients had objective partial responses involving multiple sites.	
						(5) The glucocorticoid dose was tapered by a mean of 60%	
Chronic GVHD	2014	Phase I trial	59	0.3 × 10^6^, 1 × 10^6^, or 3 × 10^6^ IU/m^2^	12-week study assessed daily treatment for 8 weeks, followed by a 4-week hiatus.	(1) Rapidly corrected the imbalance of Tcon and Treg by selectively activating Treg	^23^
						(2) Induced rapid and selective upregulation of pSTAT5 in Treg as well as the down-regulation of pSTAT5 in Tcon	
prevent GVHD	2007–2014	Phase I trial to evaluated the effects of ultra-low-dose IL-2 for GVHD Prophylaxis	16	1 × 10^5^–2 × 10^5^ IU/m^2^	3 times per week for 6 to 12 weeks	(1) CD4^+^ CD25^+^ FoxP3^+^ Tregs increased from a mean of 4.8 to 11.1%	^24^
						(2) No expansion of CD8+memory Teffs or NK cells	
						(3) No grade 3/4 toxicities were associated with ULD-IL-2	
						(4) No IL-2 patients developed grade II-IV aGVHD	
						(5) Less viral infections than comparator group	
cGVHD	2007–2014	Phase I trial to evaluated therapeutic potential of low-dose IL-2 in a chronic GVHD patient	1	1 × 10^6^ IU/m^2^	Daily administration for 8 weeks, (18 weeks hiatus, follow-up administration for another 8 weeks)	(1) CD4^+^ Foxp3^high^CD127^low^ cells expanded up to 37.3%.	^25^
						(2) Improved Treg/Th17 ratios	
						(3) Well-tolerated	
						(4) Partial response of the skin lesions and erythema in the abdomen and groin	
cGVHD	2016	Phase 2 study	35	1 × 10^6^ IU/m2	Daily subcutaneous for 12 weeks (4 weeks hiatus, follow-up administration for responders)	(1) Treg (>5-fold) and natural killer cell (>4-fold)counts rose	^26^
						(2) The Treg:Tcon ratio rose >5-fold	
						(3) 61% patients had clinical responses at multiple cGVHD sites	
						(4) Well-tolerated	
Alopecia areata	2012–2013	To evaluate the effects low-dose IL-2 in the treatment of severe Alopecia Areata	5	1.5 MIU/d then 3 MIU/d	Four 5-day courses	(1) Notable increase in Treg cell count in 4 of the 5 patients.	^27^
						(2) A concomitant decrease of the CD8+ infiltrate was observed	
						(3) No serious adverse event was reported	
						(4) Of 5 patients, 4 had a regrowth of scalp hair	
SLE	2014	To evaluate the effects of low-dose IL-2-therapy in one SLE patient refractory to standard therapies	1	1.5 MIU/d then 3 MIU//d	Four 5-day courses	(1) Treg increase	^28^
						(2) Decreased of anti-dsDNA antibody levels	
						(3) Well tolerated	
						(4) Major clinical improvement	
SLE	2016	To evaluate the effects of low-dose recombinant human IL-2 (rhIL-2) in SLE patients	40	1 MIU every other day	2 weeks, followed by a 2-week break	(1) Increase the number of CD25^high^CD127^low^ Treg cells and their function	^29^
						(2) The ratio of (TFH + TH17) cells/Treg cells’ fell significantly	
						(3) No serious adverse events were observed	
						(4) All 38 patients showed decreased disease activity	

## Conclusion

The data summarized in this review have indicated that IL-2 plays a key role in promoting the development, homeostasis and function of regulatory T cells though IL-2/STAT5 signals. Additionally, the independent clinical trials have also shown the safety of low-dose IL-2 treated in multiple autoimmune diseases. Moreover, these trials have provided preliminary indications of significant biological and clinical efficacy. We hypothesize that low-dose IL-2 may be a prime candidate therapy for other autoimmune diseases such as Rheumatoid Arthritis (RA) in which a deficiency in Treg cell number or function is present. However, these new therapeutic approaches present some undesirable adverse effects. Three potential ways to prevent these adverse effects include: (1) using IL-2/anti-IL-2 complexes to target CD25^−^ expressing cells and expand Treg without inducing IL-2-mediated expansion of autoreactive effector T cells^80^; (2) combining IL-2 administration with a blockade of cytokine pathways to promote Treg function and prevent Tfh cell development.^80^ For example, co-administration of low-dose IL-2 (or IL-2/anti-IL-2 complexes) together with anti-IL6R might synergize to promote Treg-mediated suppression^81^ in the absence of effector T-cell activation. (3) Modifying IL-2. Bell et al.^82^ compared the clinically approved IL-2 molecule, Proleukin, with two engineered IL-2 molecules with long half-live sowing to their fusion in monovalent and bivalent stoichiometry to a non-FcRγbinding human IgG1 and demonstrate that single ultra-low doses of IL-2 fusion proteins induce a prolonged state of in vivo activation that increases Tregs for an extended period of time similar to multiple-dose Proleukin. Therefore, additional well-controlled clinical trials are needed to validate improved and safer dosing strategies for low-dose IL-2 used in autoimmune diseases, but also future studies need to be done both in experimental animal models and later in clinics in order to validate IL-2/anti-cytokine therapies or IL-2/anti-IL-2 complex or IL-2 modification therapies as well.
